# Supplementary figures and images for: Dissection of the Complex Transcription and Metabolism Regulation Networks Associated with Maize Resistance to Ustilago maydis
Source: Genes (Basel). 2021 Nov 12;12(11):1789. doi: 10.3390/genes12111789 (PMC8619255; doi:10.3390/genes12111789)

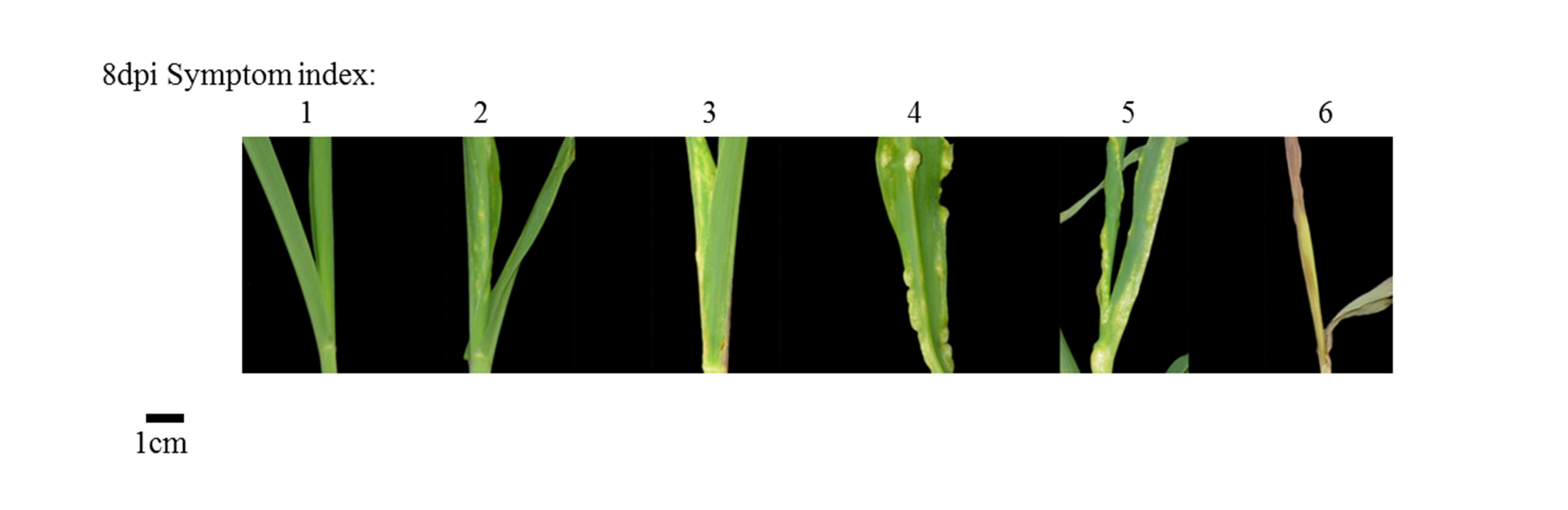

Supplement: Supplementary file 1 [file genes-12-01789-s001.zip › Supplementary Figure S1.tif]

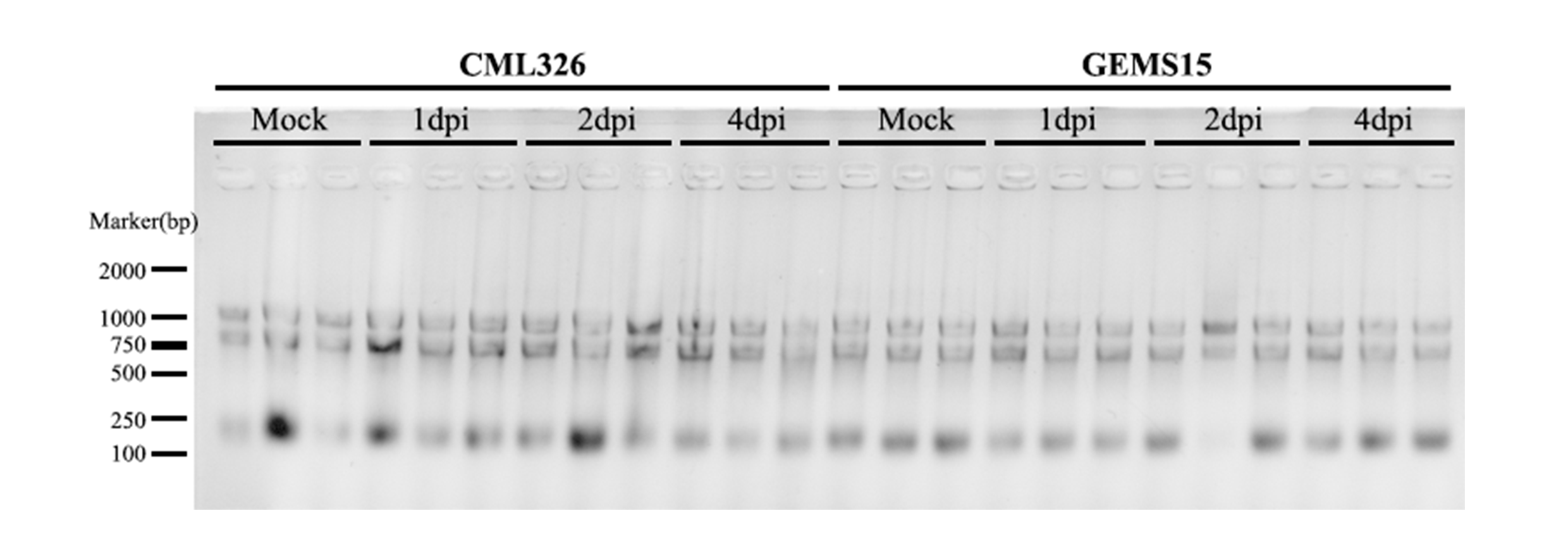

Supplement: Supplementary file 1 [file genes-12-01789-s001.zip › Supplementary Figure S2.tif]

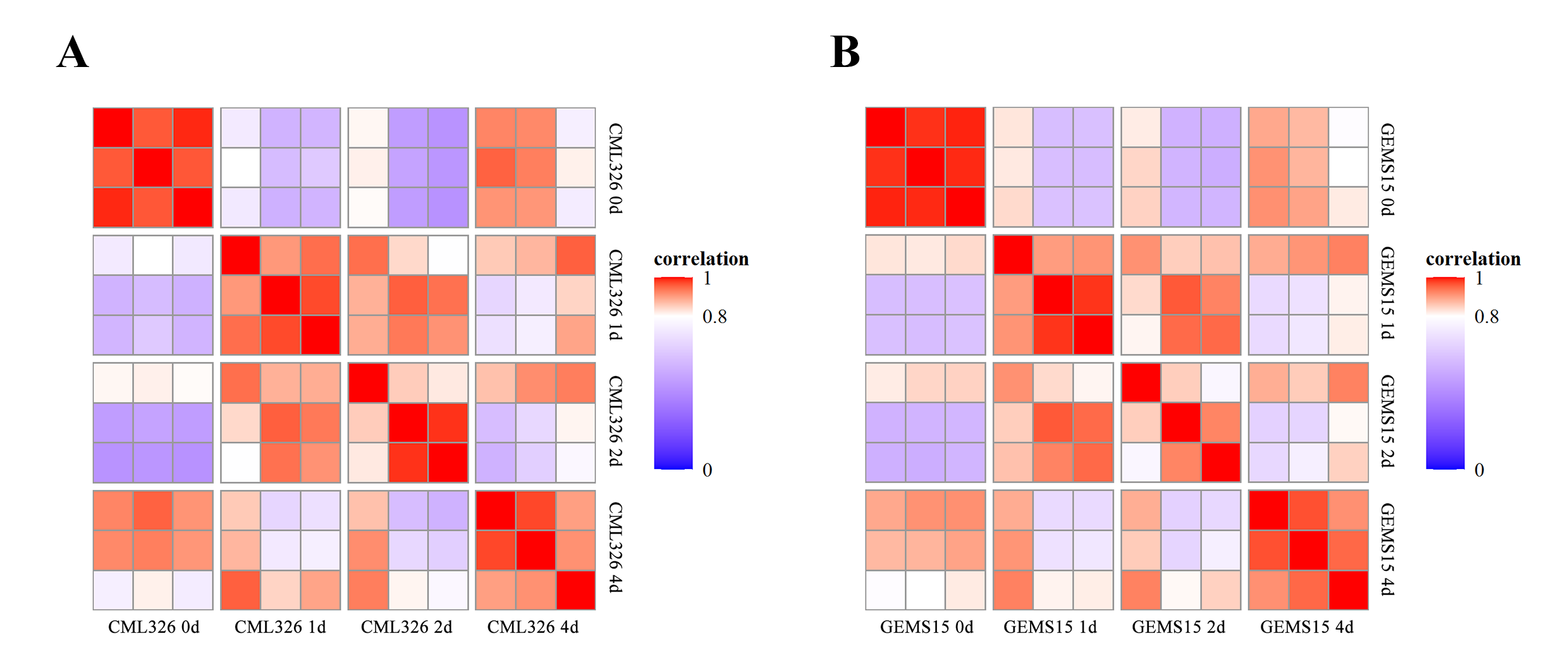

Supplement: Supplementary file 1 [file genes-12-01789-s001.zip › Supplementary Figure S3.tif]

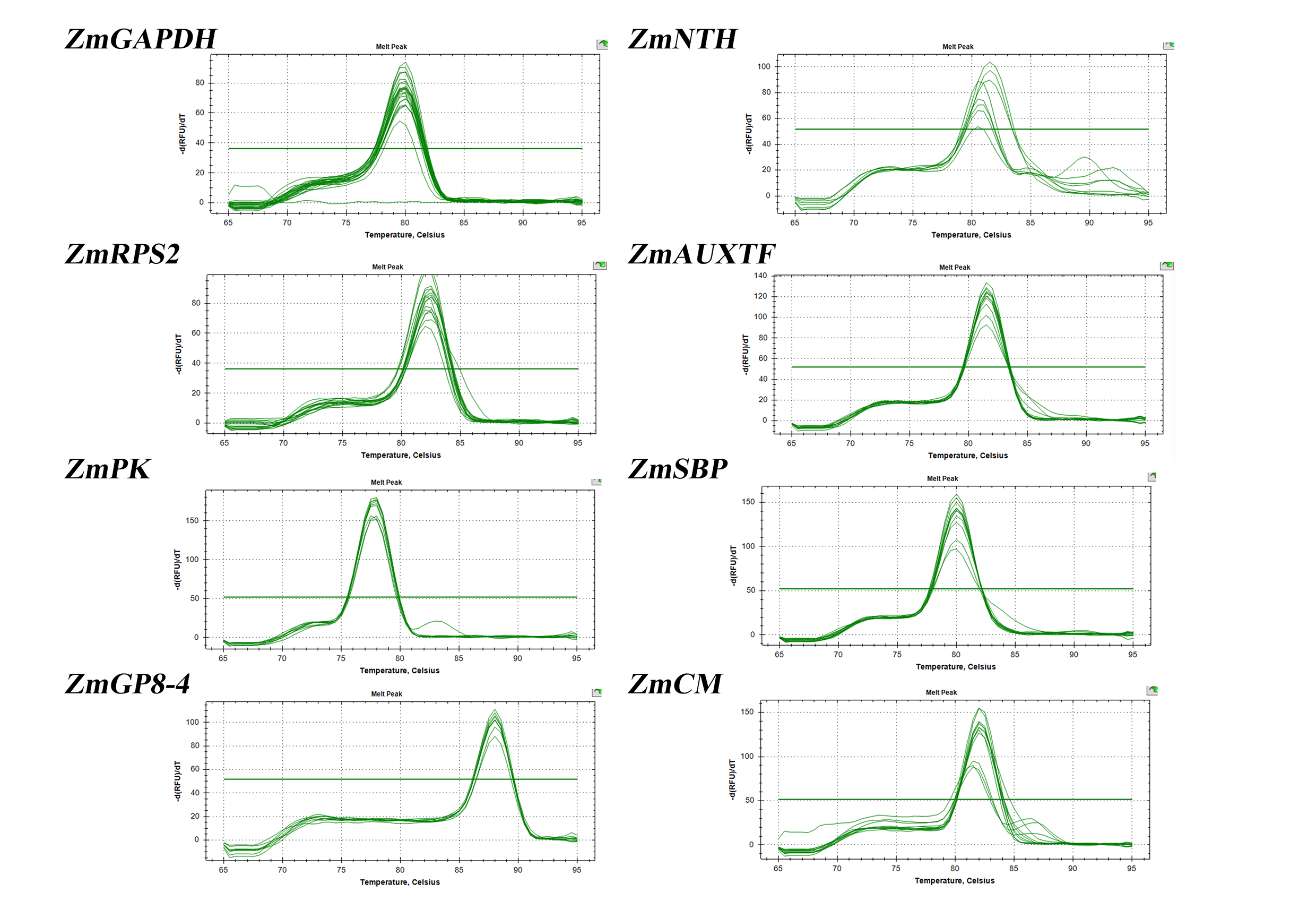

Supplement: Supplementary file 1 [file genes-12-01789-s001.zip › Supplementary Figure S4.tif]

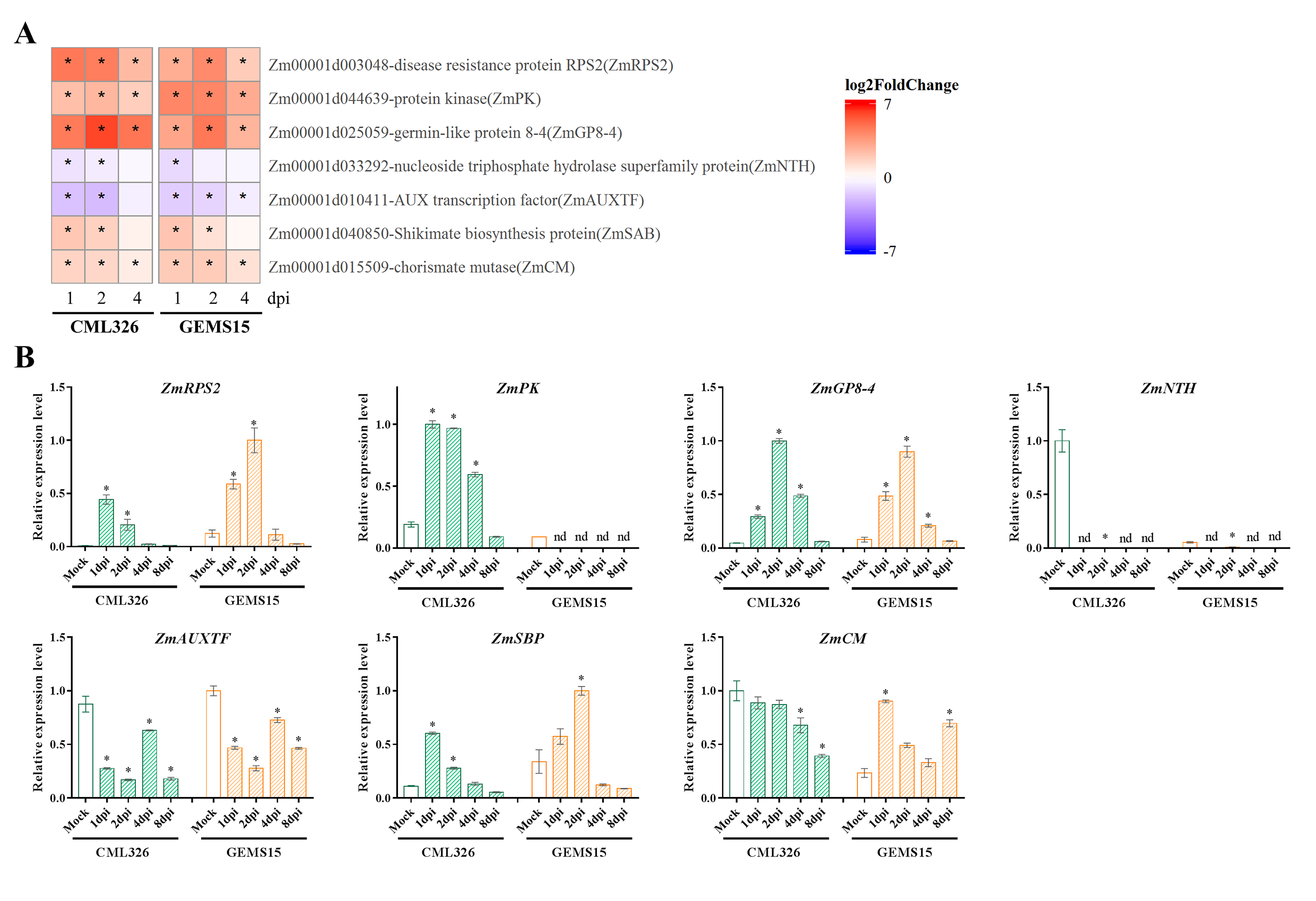

Supplement: Supplementary file 1 [file genes-12-01789-s001.zip › Supplementary Figure S5.tif]

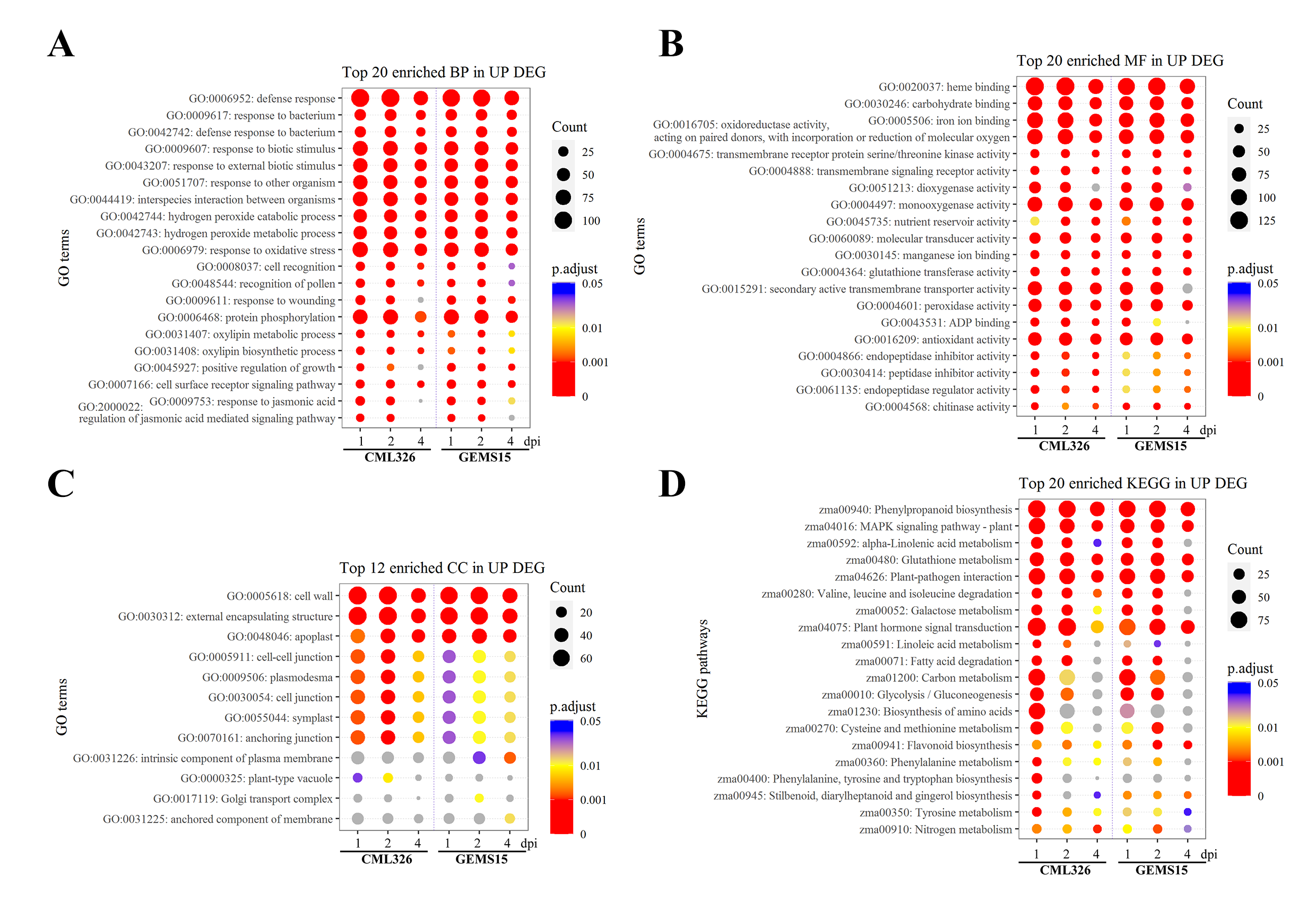

Supplement: Supplementary file 1 [file genes-12-01789-s001.zip › Supplementary Figure S6.tif]

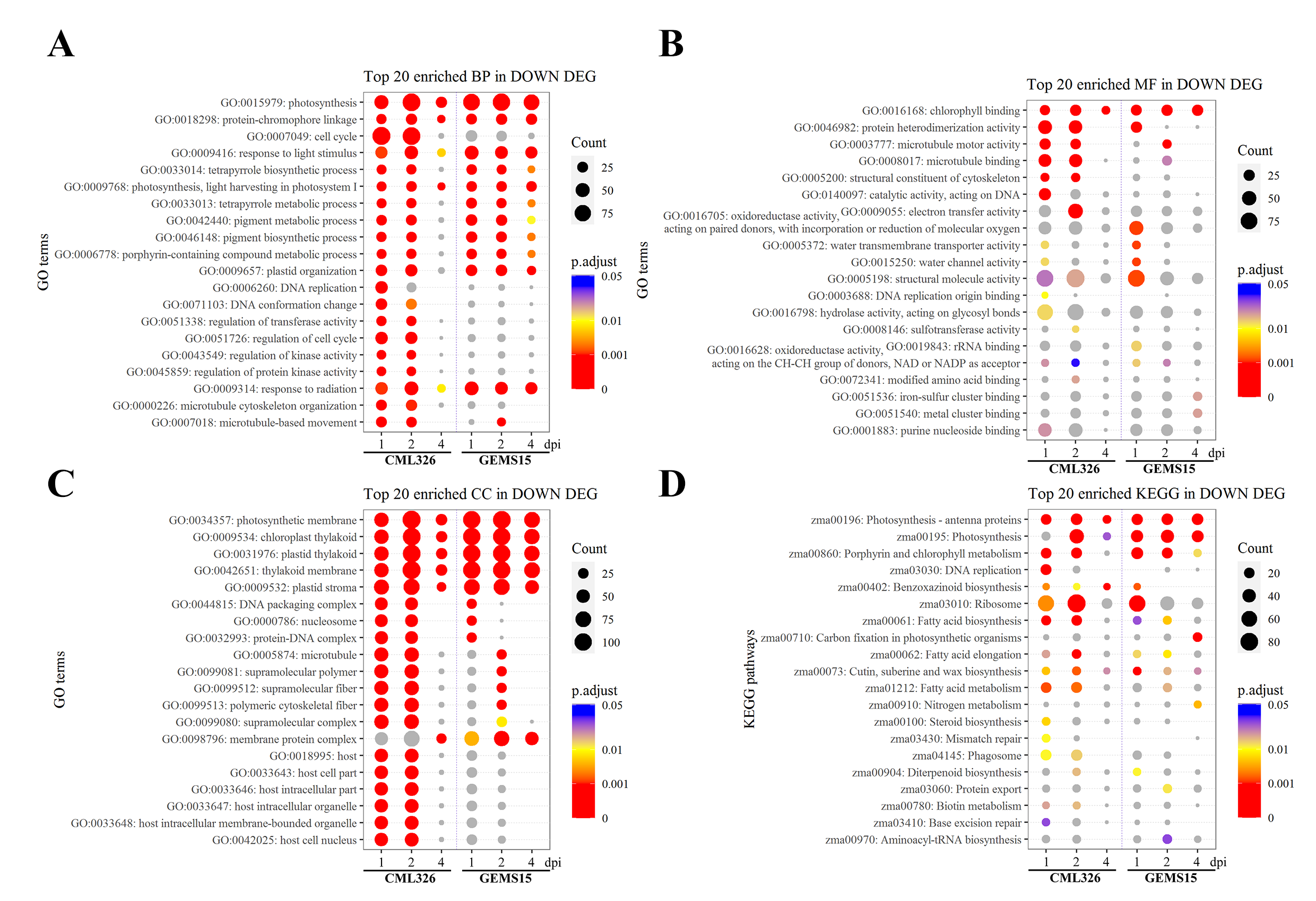

Supplement: Supplementary file 1 [file genes-12-01789-s001.zip › Supplementary Figure S7.tif]

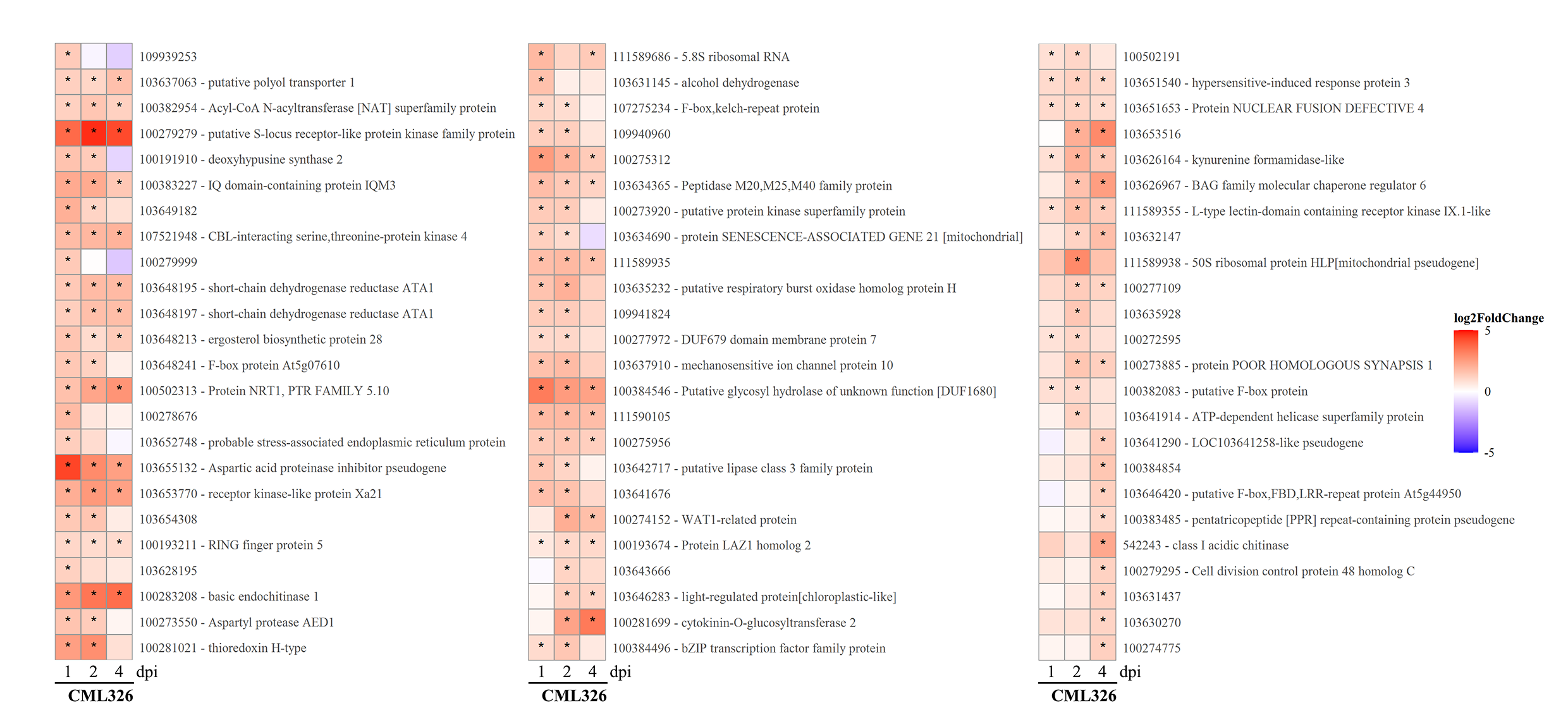

Supplement: Supplementary file 1 [file genes-12-01789-s001.zip › Supplementary Figure S8.tif]

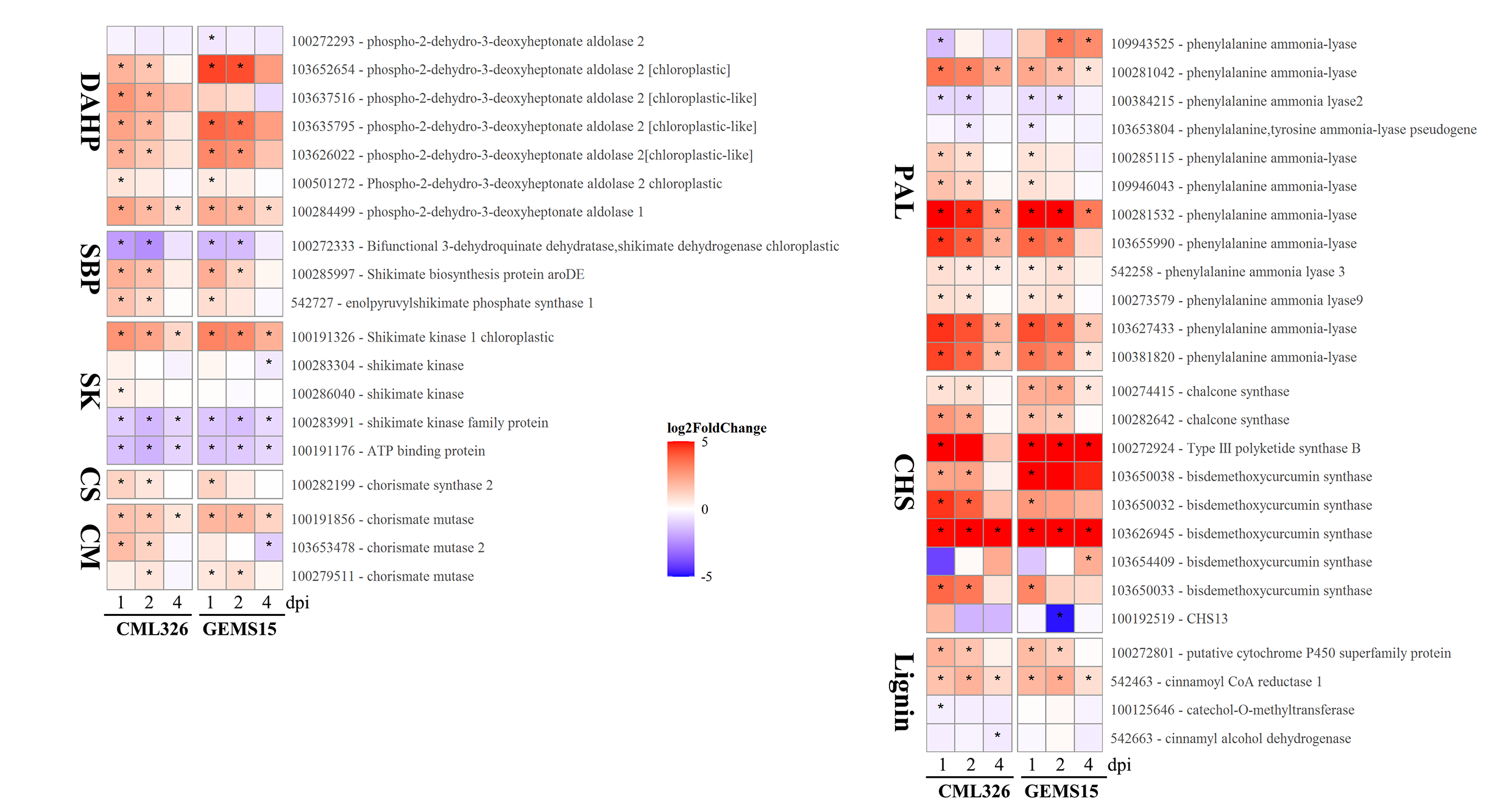

Supplement: Supplementary file 1 [file genes-12-01789-s001.zip › Supplementary Figure S9.tif]
